# A Systematic Review: The Role of Resident Memory T Cells in Infectious Diseases and Their Relevance for Vaccine Development

**DOI:** 10.3389/fimmu.2018.01574

**Published:** 2018-07-09

**Authors:** Visai Muruganandah, Harindra D. Sathkumara, Severine Navarro, Andreas Kupz

**Affiliations:** ^1^Centre for Biosecurity and Tropical Infectious Diseases, Australian Institute of Tropical Health and Medicine, James Cook University, Cairns, QLD, Australia; ^2^QIMR Berghofer Medical Research Institute, Brisbane, QLD, Australia

**Keywords:** resident memory T cells, infectious diseases, vaccine development, immunity, microbiology

## Abstract

**Background:**

Resident memory T cells have emerged as key players in the immune response generated against a number of pathogens. Their ability to take residence in non-lymphoid peripheral tissues allows for the rapid deployment of secondary effector responses at the site of pathogen entry. This ability to provide enhanced regional immunity has gathered much attention, with the generation of resident memory T cells being the goal of many novel vaccines.

**Objectives:**

This review aimed to systematically analyze published literature investigating the role of resident memory T cells in human infectious diseases. Known effector responses mounted by these cells are summarized and key strategies that are potentially influential in the rational design of resident memory T cell inducing vaccines have also been highlighted.

**Methods:**

A Boolean search was applied to Medline, SCOPUS, and Web of Science. Studies that investigated the effector response generated by resident memory T cells and/or evaluated strategies for inducing these cells were included irrespective of published date. Studies must have utilized an established technique for identifying resident memory T cells such as T cell phenotyping.

**Results:**

While over 600 publications were revealed by the search, 147 articles were eligible for inclusion. The reference lists of included articles were also screened for other eligible publications. This resulted in the inclusion of publications that studied resident memory T cells in the context of over 25 human pathogens. The vast majority of studies were conducted in mouse models and demonstrated that resident memory T cells mount protective immune responses.

**Conclusion:**

Although the role resident memory T cells play in providing immunity varies depending on the pathogen and anatomical location they resided in, the evidence overall suggests that these cells are vital for the timely and optimal protection against a number of infectious diseases. The induction of resident memory T cells should be further investigated and seriously considered when designing new vaccines.

## Introduction

Traditionally, memory T cells have been subdivided into two broad categories: effector memory and central memory T cells (T_EM_ and T_CM_, respectively). After the realization that some memory T cells fail to egress out of peripheral tissues back into the blood stream, it became clear that this dichotomous distinction of memory T cells did not account for the complete diversity of the memory T cell population. This led to the discovery of a third subset of memory T cell. Appropriately, dubbed “Tissue-resident memory T cells” (here after referred to as T_RM_), this newly defined population exhibits the unique feature of remaining localized in peripheral tissues (1). As such, these cells provide enhanced localized immunosurveillance and protection of peripheral tissues when compared to T_EM_ and T_CM_. T_RM_ have been characterized in many peripheral tissues, including skin, lungs, brain, liver, the female reproductive tract, and the gastrointestinal mucosa. Given the huge variance in their location of residence, this subset of memory cell is highly heterogeneous, phenotypically varying depending on their anatomic location and the inflammatory cues produced by their respective microenvironment. Although experimental techniques such as parabiosis can definitively distinguish T_RM_ from circulating memory T cells, other less complex methods of identifying T_RM_ are more frequently used. The co-expression of CD69 and CD103 is commonly used as a marker of tissue residence, although it appears not all bona fide T_RM_ are defined by this particular phenotype. Regardless, T_RM_ have been implicated in a wide range of physiological functions, such as providing protection against pathogens and cancerous cells, as well as in many pathological states such as autoimmune and other inflammatory diseases. The exploration of T_RM_ biology and the role they play in maintaining homeostasis has broad implications for human health. Currently, our understanding of T_RM_ function is largely constrained within the context of infectious diseases. As of now, it appears that T_RM_ are better adapted to providing rapid protection against pathogen invasion when compared to their circulating counter parts (2–4). Thus, vaccines of the future would ideally establish a population of protective T_RM_ at the portals of entry most at risk of pathogen invasion to provide immediate and effective immunity, rather than relying on the delayed recruitment of effector cells from the circulating pool of memory cells. Since parenterally administered vaccines induce minimal tissue-specific protection, current routes of administering vaccines may need to be revised (5, 6). The present review will primarily focus on the role of T_RM_ in the immune response generated to a range of human pathogens and discuss future avenues for the development of T_RM_-based vaccines.

## Methodology

**Figure 1:**
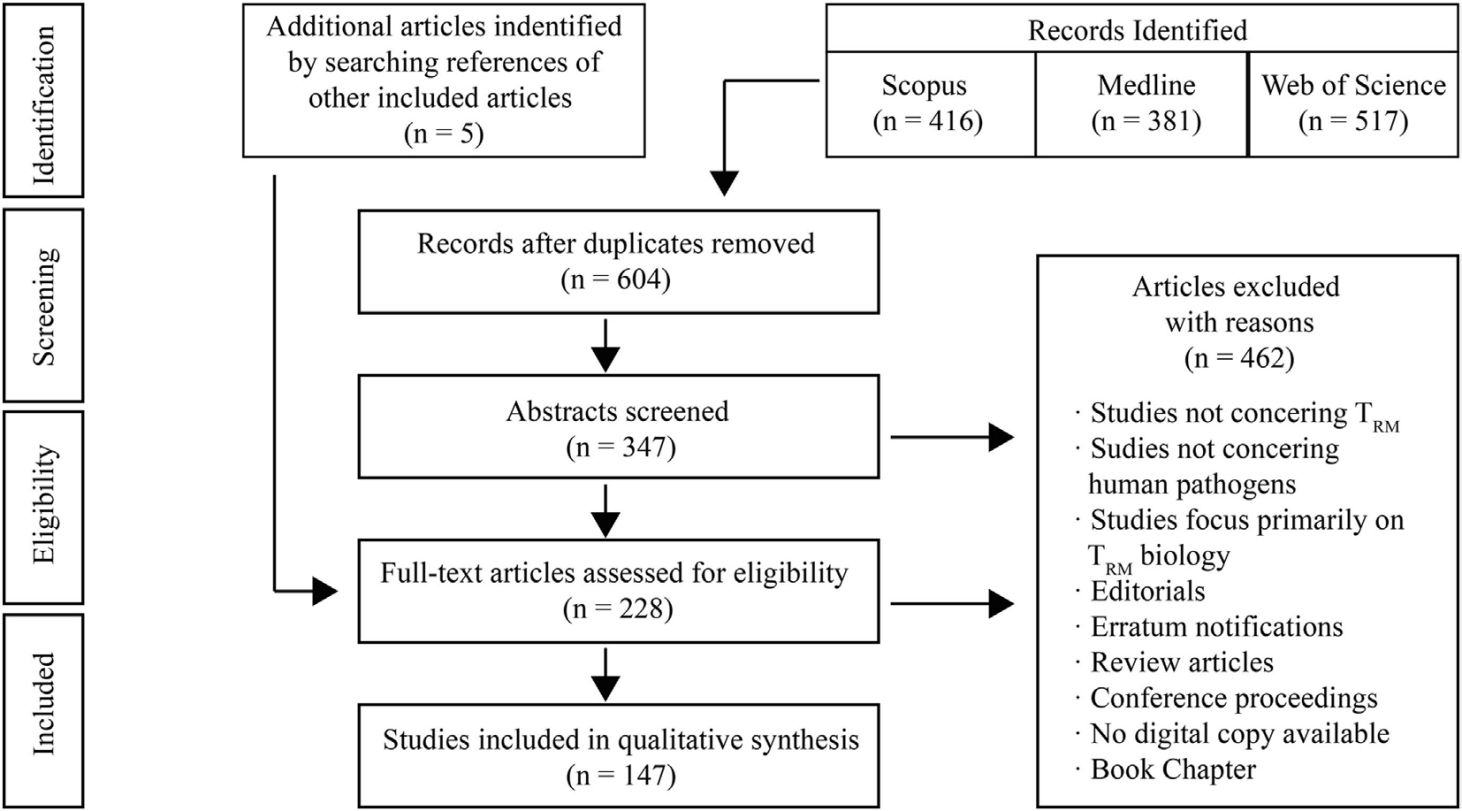
Literature search strategy. The search strategy used revealed 381 records in Medline (Ovid), 416 in SCOPUS, and 517 in Web of Science. This resulted in a total number of 1,314 records. After removing duplicates, there were 604 records. Screening of titles resulted in the exclusion of 257 records, as they did not address resident memory T cells, human infectious diseases, or neither. Others records were excluded as they were reviews, editorials, meeting abstracts, book chapters, poster presentations, or erratum notifications. The abstracts of the remaining 347 records were analyzed and a further 124 publications were excluded due to their focus on T_RM_ biology. The full texts of the remaining studies were reviewed. 81 of these texts were excluded for aforementioned reasons. Co-authors were consulted when there was ambiguity regarding the relevance of a study. In total, 142 publications from the search were included. 5 additional studies were included by screening the references of studies from the search results and following external review.

A systematic search of published literature was conducted. Literature was critically evaluated for evidence of the role T_RM_ play during infections and in vaccinology. A flowchart summarizing our methodology has been included (Figure 1). The preparation of this review was guided by the *PRISMA-P 2015 guideline* (7).

Final searches of literature were performed on March 23, 2018 in Medline, SCOPUS and Web of Science by the first author. The Boolean search strategy used was as following (“resident memory t cell*” OR “t resident memory cell*” OR “tissue resident memory cell*” OR “resident memory” OR “tissue memory”). The references of included studies were also screened for other relevant publications.

Both human and animal studies that use surface markers of residence or other established techniques such as intravascular staining and parabiosis to illustrate localization of T cells to peripheral tissues, as well as T cell phenotyping were included. Studies were also screened for their relevance to human pathogens, and thus animal infection models that are analogous to human infectious diseases were included. Studies were included irrespective of published date. Only published and accepted manuscripts of original research were included. Publications that primarily focused on T_RM_ biology (ontogeny, cellular metabolism, etc.) or non-infectious diseases were not included. Certain non-communicable diseases such as hepatocellular carcinoma and cervical cancer that can be caused by pathogens are briefly mentioned within the broader discussion of T_RM_-mediated immunity.

## Results of Search

The results of the search strategy are summarized in Figure 1.

## Data Synthesis and Analysis

**Figure 2:**
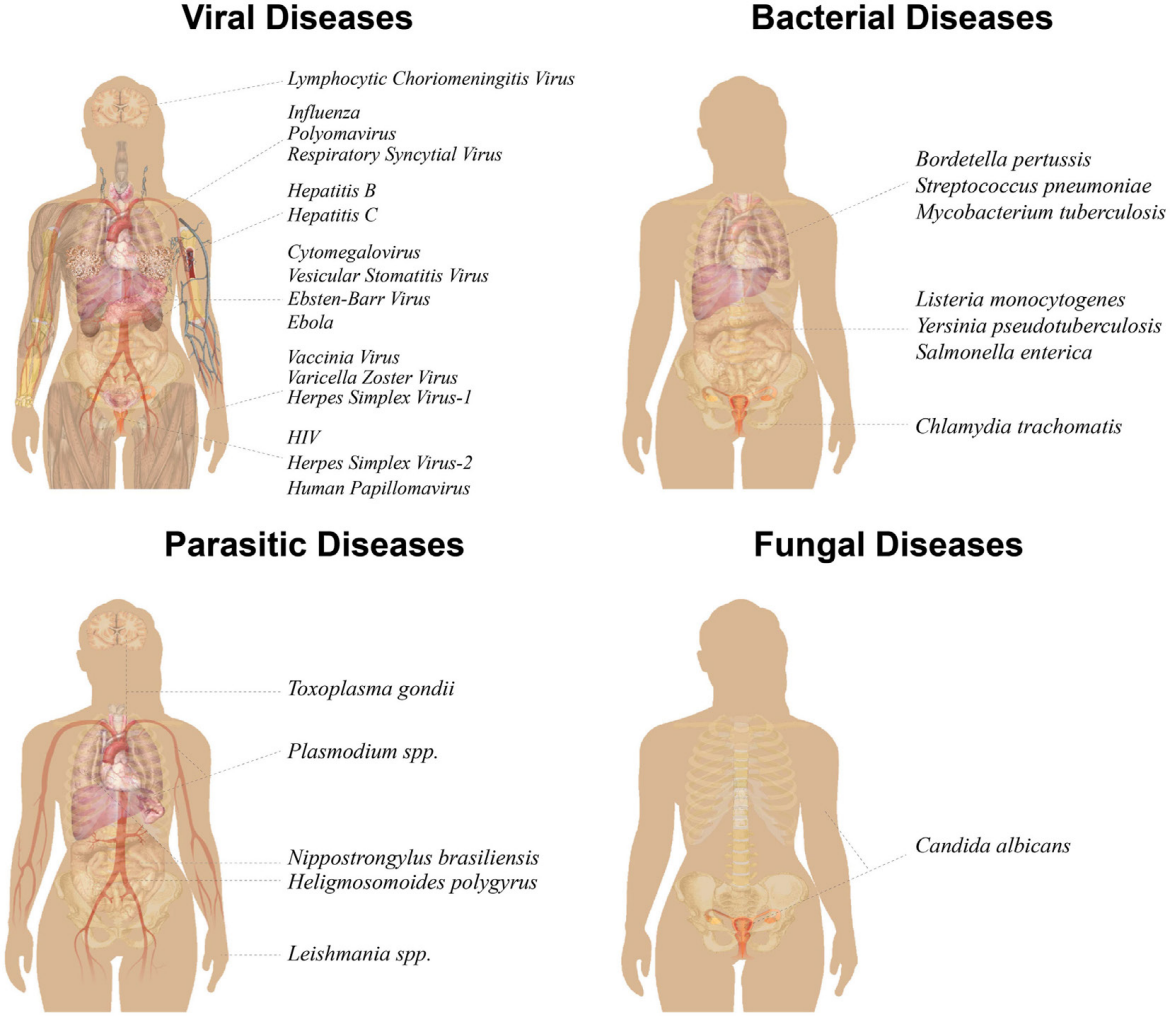
Illustration of human-relevant diseases for which a role of T_RM_ has been reported. T_RM_ have been studied in 16 viral diseases (top left), seven bacterial diseases (top right), five parasitic diseases (bottom left), and one fungal disease (bottom right). Pathogens have been grouped according to organs or organ systems that have been studied in the context of T_RM_, including the respiratory, gastrointestinal, and urogenital tracts, as well as brain, skin, liver, lymphatics, and circulation. *Nippostrongylus brasiliensis* and *Heligmosomoides polygyrus* are mouse pathogens for human *Necator americanus* and gastrointestinal helminth infections, respectively. Image modified from https://commons.wikimedia.org/wiki/File:Female_shadow_anatomy_without_labels.svg.

The first author conducted extraction of data from relevant studies. This review has been divided into sections based on pathogen type: viruses, bacteria, parasites/helminths, and fungi (Figure 2). The studies included in this review contain the most relevant findings related to immune responses generated by T_RM_ against human pathogens, or make use of novel strategies for T_RM_ generation. We apologize to authors whose work could not be included in this review.

## The Role of T_RM_ in Viral Infections

As of present, T_RM_ immune responses are by far mostly studied in the context of viral pathogens. The following section will present findings by specific viral pathogen/viral disease.

## Herpes Simplex Virus (HSV)

Herpes simplex virus causes infections that present with a varying range of symptoms. The primary clinical manifestations of HSV infection are intraepithelial vesicles. There are two antigenically distinct HSV subtypes: HSV-1 and HSV-2, causing cold sores and genital warts, respectively (8). However, both sub-types can be the etiology of either clinical disease as sexual transmission allows for spread between the two sites (9). Both viruses establish a life-long latent infection within the surrounding nervous tissue, and control of HSV infection requires effective cell-mediated immune responses to prevent reactivation. However, co-morbid illnesses, immunosuppressive drugs, UV exposure, and psychological stress can hinder immune control. A number of studies suggest that T_RM_ are implicated in controlling HSV-1 latency in the trigeminal ganglia (10). HSV-1 infection models primarily focus on infections of the skin and nervous tissue such as the trigeminal ganglia and the eyes (11–22). Following acute infection with HSV-1, CD8^+^ T_RM_ remain localized to the skin initially infected and are also found surrounding latently infected sensory ganglia (11). However, evidence suggests that multiple exposures to cognate antigen can substantially increase the T_RM_ population in not only the site of HSV infection, but also in distant skin (12). CD8^+^ skin T_RM_ appear to resemble the antigen-presenting Langerhans cells of the skin, extending dendritic projections into the surrounding tissue, probably in an attempt to survey the local area for antigen (13). This is supported by evidence from confocal microscopy and intravital imaging (21) that suggests these T_RM_ can travel between keratinocytes (13). However, unlike Langerhans cells, these T_RM_ do not extend into the stratum corneum (13, 17). It also appears that skin T_RM_ are not specifically attracted to virally infected cells, and thus migrate throughout the epidermis in a random manner (17, 21). By extension, it can be inferred that skin T_RM_ may take a considerable period of time before identifying virally affected cells. As such, it may be safe to assume that a critical mass of skin T_RM_ is needed in order to afford timely protection. This notion is supported by the observation that protection appears to be dependent upon the local density of T_RM_ (17). Upon antigen recognition, skin T_RM_ undergo a change in their morphological and motility pattern, decelerating their migratory rate and losing their dendricity (13, 17). This is probably indicative of a shift in role from immunosurveillance to effector function. Furthermore, the maintenance of HSV-1-specific T_RM_ populations appears to be independent of circulating T cells in both skin and trigeminal ganglia (14, 17). Skin T_RM_ appear to be able to sustain their numbers through local proliferation after secondary infection (17). However, after a combined corticosterone and stress-induced reduction of trigeminal ganglia CD8^+^ T cells (presumably T_RM_), there appeared to be no increased proliferation of remaining T cells when compared to the homeostatic proliferation rates as indicated by BrdU incorporation (14). Analysis of HSV-1-specific skin and dorsal root ganglia T_RM_ during acute immunity and later time points revealed that transcription of cytolytic molecules decreases with time. As such, T_RM_-based immunity in the long term may not be reliant on enhanced cytolytic effector functions, but rather on the localization of these cells at sites susceptible to reinfection (15). The chronic inflammatory response induced by persistent viral gene expression during latency leads to T_RM_ exhaustion in the brain ependymal region, rendering them unable to control HSV-1 infection (16). Perhaps the reason why T_RM_ downregulate their cytolytic genes during times of homeostasis is because continuous expression may lead to exhaustion. Nevertheless, it appears that the generation of CD8^+^ T_RM_ in skin and ganglia may be a viable option for protection against HSV-1 infection or reactivation. Local inflammation of the skin and mucosa alone can encourage the recruitment of T_EM_ to these peripheral sites where differentiation into the T_RM_ phenotype occurs. This was demonstrated using 2,4-dinitrofluorobenzene, a contact-sensitizing agent. Furthermore, the application of nonoxynol-9 (a spermicide agent) to the female genital tract enhanced protection against HSV challenge, correlating with higher numbers of CD103^+^ T cells localizing to the epithelium (19). Hence, agents that can be applied to specific tissue and that cause a localized, general inflammatory response may be a strategy worth exploring for the generation of T_RM_. More specifically, however, the CXCL10/CXCR3 chemokine pathway appears to be vital in generating T_RM_, as mice deficient in either CXCL10 or CXCR3 were unprotected against HSV-1 UV-B light-induced reactivation challenge. Furthermore, the administration of CXCL10 into deficient mice through the use of a neurotropic virus vector amplified T_RM_ in the trigeminal ganglia, conferring better protections against reactivation challenge (20). CXCL10 administration through the use of a rAAV8-CamKIIa-GFP-CamKIIa-CXCL10 vector showed similar results (18). Samples from human patients that were asymptomatic but seropositive for HSV-1 infection were used to determine which 467 HLA-A*0201-restircted CD8^+^ T cell epitopes were immunodominant in the HSV-1-specific immune response. These asymptomatic individuals generated a high number of polyfunctional CD8^+^ T_EM_ against three epitopes. HLA-A*0201 transgenic mice were primed with these epitopes and subsequently treated with an ocular topical preparation containing rAAV8-CamKIIa-GFP-CamKIIa-CXCL10 to deliver exogenous CXCL10 chemokine. Results from UV-B reactivation challenge demonstrated that this strategy was able to reduce viral shedding in tears and recurrent herpetic ocular disease (18). This strategy may be beneficial in rationally developing novel vaccines against other diseases.

Human studies have also been conducted in HSV-2 infection. Samples from the genital tract of HSV-2-infected women contained populations of HSV-2-specific T cells with a T_RM_ phenotype (23). More interestingly, a population of CD8αα^+^ T_RM_ that reside at the dermal–epidermal junction have also been described in biopsies of HSV-2-infected humans. This unique positioning suggests that these cells may be able to survey the neural tissue from which virus travels to the skin during reactivation (24). Thus, T_RM_ play a role in the natural immune response against HSV-2 infection. Although it was already demonstrated that T cells could be recruited to peripheral tissues using inflammatory agents (19), the “prime and pull” vaccine strategy was first described in a HSV-2 infection model (25). In this study, the investigators explored the novel idea of parenterally immunizing mice and subsequently topically administering CXCL10 into the vagina before challenging with HSV-2. Mice that underwent the prime and pull protocol showed minimal signs of clinical disease and had a survival rate of 100%. Naive and parenterally immunized mice that did not receive the pull treatment developed clinical disease and exhibited high mortality rates. This strategy also demonstrated the capacity to prevent infection of sensory neurons (25). Further investigation of this protocol revealed that immunity was largely dependent on INF-γ produced by CD8^+^ T_RM_ (26). Re-stimulation of this CD8^+^ T_RM_ was dependent on a population of CD301b^+^ dendritic cells that resided in the lamina propria. In fact, depletion of CD301b^+^ dendritic cells using a diphtheria toxin model rendered the prime and pull strategy ineffective and mice suffered high morbidity and mortality rates (26). Although a non-specific inflammatory stimulus such as nonoxynol-9 may be sufficient to pull CD8^+^ T cells in to the female reproductive tract and subsequently convert them to T_RM_, it appears that antigen presentation by CD301b^+^ dendritic cells is needed for CD8^+^ T_RM_-mediated immunity at this site. A different study that made use of a topical vaccine containing a human papillomavirus (HPV) vector expressing gB and gD ectodomains of HSV-2 has shown the capacity to generate T_RM_ in the reproductive tract, and reduce viral shedding and clinical disease (27). This study highlights the capacity of HPV vectors to induce T_RM_ in the genital tract, a vaccine strategy that may be applicable to other sexually transmitted infections. HSV-2-specific CD8^+^ T_RM_ can also be generated using a “chemical-free and biological-free” laser adjuvant, a protocol that could be explored in other infectious models (28). While the vast majority of studies have assessed the protective capabilities of CD8^+^ T_RM_ during HSV infection, very few studies have analyzed the role of CD4^+^ T_RM_ in HSV infections (21, 29). Intravaginal vaccination of mice with thymidine kinase negative HSV-2 (an attenuated form of the virus) provided full protection against challenge with wild-type HSV-2, independent of CD8^+^ T cells and B cells. Instead, parabiosis studies demonstrated that CD4^+^ T_RM_ are required within the genital tract mucosa for immunity in this model. These CD4^+^ T_RM_ are polyfunctional, secreting IFN-γ, TNF-α, and IL-2, and resided in organized, non-tertiary immune structures called memory lymphocyte clusters (MLCs). These MLCs appear to assemble under the influence of macrophage-secreted CCL5. Upon antigen stimulation, the CD4^+^ T_RM_ within the MLCs expand and secrete high levels of IFN-γ. The investigators of this study also report that circulating memory T cells were “barely recruited” when MLCs were present in the mucosa. This suggests that CD4^+^ T_RM_ may be capable of clearing or controlling infection even before recruiting signals are generated in a magnitude large enough to attract circulating T cells to the site of infection (29). It still remains necessary to explore whether a critical mass of CD4^+^ T_RM_-containing MLCs are needed within the genital tract to provide protection. It is likely that this profound role of CD4^+^ T_RM_ in mediating immunity during HSV-2 infection is due to the location of the infection (genital tract) rather than the viral factors alone. This is supported by the fact that MLCs have also been found in the genital tract of both human and mice secondary to infections caused by a bacterial pathogen [refer to *Chlamydia trachomatis* (*Ct*) section of this review]. Despite the difficulties in generating CD4^+^ TRM following prime and pull vaccination, an ideal vaccine against HSV-2 should generate CD8^+^ and CD4^+^ T_RM_ (25).

## Influenza

Influenza viruses are a major cause of respiratory infections. Although influenza vaccines have been in use for many years, antigenic drift of surface hemagglutinin and neuraminidase proteins require annual immunizations. Antigenic shift can result in highly virulent strains of influenza that cause devastating pandemics (30). The ideal influenza vaccine would provide heterotypic immunity that prevents the escape of newly mutated viruses. Current influenza vaccines rely on generating high neutralizing antibody titers to protect against infection. Although this strategy has demonstrated efficacy in mediating protection, the inability of antibodies to neutralize new variants of the virus has sparked research into alternate strategies (31). Growing evidence suggests that efforts should be focused on developing vaccines that generate T_RM_-mediated immunity (32). Analysis of human samples has revealed that influenza-specific T_RM_ can be found in substantial numbers in lung tissue, highlighting their role in natural infection (33, 34). Despite expressing low levels of granzyme B and CD107a, these CD8^+^ T_RM_ had a diverse T cell receptor (TCR) repertoire, high proliferative capacities, and were polyfunctional (34). Influenza infection history suggests a greater level of protection against re-infections likely due to the accumulation of CD8^+^ T_RM_ in the lungs (35). Furthermore, the natural immune response to influenza A virus infection in a rhesus monkey model demonstrated that a large portion of influenza-specific CD8^+^ T cells generated in the lungs were phenotypically confirmed as CD69^+^CD103^+^ T_RM_ (36). Unlike lung parenchymal T_RM_, airway CD8^+^ T_RM_ are poorly cytolytic and participate in early viral replication control by producing a rapid and robust IFN-γ response (37, 38). Bystander CD8^+^ T_RM_ may also take part in the early immune response to infection through antigen non-specific, NKG2D-mediated immunity (39). The generation of functional T_RM_ that protect against heterosubtypic influenza infection appear to be dependent on signals from CD4^+^ T cells (40). A role for CD4^+^ T_RM_ has also been reported (41). Much like their CD8^+^ counterparts, CD4^+^ T_RM_ also produce a significant IFN-γ response during early infection (42, 43). Aside from the CD8^+^ and CD4^+^ subsets of T_RM_, a subset of NK1.1^+^ double negative T memory cells which reside in the lungs also play a role in influenza infection (44). Taken together, these studies and others (45–47) demonstrate that T_RM_ are required for optimal protection. However, unlike T_RM_ in other locations, such as the skin, lung T_RM_ are not maintained for extended periods of time. This gradual loss of lung T_RM_ appears to be the reason for the loss in heterotypic immunity against influenza infection (45, 46, 48). Lung T_RM_ exhibit a transcriptional profile that renders them susceptible to apoptosis (48). Despite conflicting evidence (49), it appears that maintenance of the lung CD8^+^ T_RM_ populations relies on the continual seeding from circulating CD8^+^ T cells. However, with time, circulating CD8^+^ T cells adopt a transcriptional profile that reduces their capacity to differentiate into T_RM_. Expanding the CD8^+^ T_EM_ compartment through booster vaccination may circumvent the problem of these time-sensitive transcriptional changes (48). There is also conflicting evidence regarding the requirement of local antigen for the generation of T_RM_ within the lung (48, 50). Continuing to find ways to generate and maintain lung T_RM_ is of great importance for vaccines against pulmonary infections. Intranasal administration of vaccines seems to encourage the development of a strong mucosal immune response (51, 52). Intranasal administration of Live Attenuated Influenza Vaccine (FluMist) in a mouse model induced both CD4^+^ and CD8^+^ T_RM_ that provided a degree of cross-strain protection independent of T_CM_ and antibodies (53). The intranasal administration of a PamCys2 or Adjuplex has demonstrated capacity for producing protective influenza-specific lung CD8^+^ T_RM_ in similar numbers and IFN-γ secreting potential when compared to the natural response to influenza infection (54, 55). Furthermore, a vaccine containing virus-like particles with tandem repeat M2e epitopes generated heterotypic immunity through the induction of antibodies, and protection correlated with IFN-γ-secreting CD8^+^ T_RM_ (56). A Modified Vaccinia Ankara-vectored virus expressing conserved influenza nucleoprotein and matrix protein 1 elicited an IFN-γ secreting CD4^+^ T cell and CD8^+^ T_RM_ response (57). Co-administration of 4-1BBL (CD137 signal) along with an influenza nucleoprotein expressing replication defective adenovirus vector *via* the intranasal route stimulated and boosted a lung CD8^+^ T_RM_ response through the recruitment of circulating T cells (58). Intranasal administration of 4-1BBL may serve as a promising “pull” strategy in systemically primed individuals. Another potential “pull” strategy is the intranasal administration of Fc-fused IL-7. This protocol was used as a pre-treatment before influenza A infection, and demonstrated protective capacities in mice against lethal challenge. It appears that Fc-fused IL-7 recruits polyclonal circulating T cells into the lungs, which subsequently reside in the lung tissue as “T_RM_-like cells” (59). Intranasal administration of Fc-fused IL-7 after systemic priming may be able to recruit influenza-specific T cells into the lungs, and may be a strategy for inducing lung T_RM_. An antibody targeted vaccination strategy in which antigens are coupled to monoclonal antibodies against CD103^+^ or DNGR-1^+^ dendritic cells has also been shown to elicit a protective CD8^+^ T_RM_ response (47, 60).

## Human Immunodeficiency Virus (HIV)

Human immunodeficiency virus is a retrovirus that is transmitted *via* contact with infected blood and other fluids such as semen and vaginal secretions. The virus specifically targets the surface proteins CD4, CXCR4, and CCR5, with the natural progression of disease resulting in the depletion of CD4^+^ T cells. As a consequence, infected individuals are left in an immunocompromised state referred to as aquired immunodeficieny syndrome (AIDS), which is characterized by fatal opportunistic infections and malignancies. Although the development of therapeutics such as anti-retroviral therapy has reduced the incidence of AIDS, HIV/AIDS continues to contribute significantly to global morbidity and mortality. Evidence shows that CD8^+^ T cells are vital in controlling early infection (61). Studies of human tissue samples have revealed that T_RM_ are generated in response to HIV infection in multiple locations including the gastrointestinal tract and the female reproductive tract (62–65). Furthermore, individuals who appeared to naturally control infection had T_RM_ that were capable of producing the highest polyfunctional immune responses when compared to individuals who did not. However, the T_RM_ population within the HIV-specific CD8^+^ T cell compartment in individuals who controlled infection was under-represented when compared to individuals who were viremic (62). Although not confirmed, this may be due to the higher ability of polyfunctional T_RM_ in these individuals to recruit circulating T cells, thereby only altering the T_RM_ proportion. Similar to other infections in various sites, CD8^+^ T_RM_ in the context of HIV can be sub-divided into two subsets based on the expression of CD103 (62, 63). Analysis of the ectocervial epithelium and menstrual blood revealed that HIV-infected women were more likely to have CD103^−^ T_RM_ when compared to healthy individuals (63, 64). This reduced expression of CD103 may be explained by the HIV-induced depletion of CD4^+^ T cells which appear to be vital in providing help to CD8^+^ T cells for up-regulating CD103 (64). The CD103^−^ populations of the ectocervix resided closer to the basement membrane of the epithelium when compared to their CD103^+^ counterparts. Interestingly, the CD103^+^ population from infected individuals appears to express higher levels of PD-1 (63). In a separate study, adipose PD-1^+^ CD4^+^ T_RM_, appeared to remain relatively inactive during HIV infection and may serve as a reservoir for HIV (65). As such chronically activated T_RM_ and T_RM_ exposed to immunomodulated environments (such as the adipose tissue) may be unable to elicit a full effector response, favoring the progression of HIV infection. It also appears that HIV has the ability to disrupt CCR5-mediated CD8^+^ T cell migration into the cervical mucosa, thereby impairing the development of T_RM_ populations (66). Regardless, human studies suggest that T_RM_, especially CD8^+^ T_RM_, play an important role in combating HIV infection and thus may be valuable targets for vaccine development. Since the most common mode of transmission of HIV is through sexual intercourse, it may be desirable to explore strategies that induce anti-HIV CD8^+^ T_RM_ in the female and male reproductive tract and rectosigmoid epithelium. In a Simian Immunodeficiency Virus model of rhesus macaques, intravenous administration of SIVmac239Δ*nef* generated a population of CD8^+^ T_RM_ in the vaginal tissue and the gut that participated in protection (67). In a murine model, a mucosal vaccination strategy in which intranasal administration of an influenza-vector expressing the HIV-1 Gag protein p24 followed by an intravaginal booster induced CD8^+^ T_RM_ in the vagina. Antigen stimulation of these CD8^+^ T_RM_ resulted in the recruitment of B cells, natural killer cells, and CD4^+^ T cells (68). While the recruitment of innate and adaptive immune cells may be beneficial in early viral clearance, the recruitment of CD4^+^ T cells may be detrimental in the context of HIV as they are the target for HIV. Hence, incidental recruitment of CD4^+^ T cells to sites of HIV entry (female reproductive tract and rectum) by prime and pull vaccination strategies may unintentionally increase susceptibility to infection. A micro-needle array delivery system that utilizes a recombinant adenovirus vector containing the HIV-1 protein Gag, has also produced promising results in generating T_RM_. These HIV-specific T_RM_ were found in the female reproductive tract and respiratory tract of immunized mice and responded to local antigenic stimulation through expansion and production of IFN-γ and granzyme B (69). Using this micro-needle array delivery system as a priming strategy followed by intravaginal delivery of a booster concoction serving as a pull strategy may be an interesting protocol worth exploring.

## Vaccinia

Vaccinia is a poxvirus that usually causes a very mild or asymptomatic infection in immunocompetent individuals. Immunity to vaccinia virus also provides sufficient protection against smallpox, which allowed for its eradication following administration of the live vaccinia virus (70). Despite elimination, smallpox remains a priority on the global agenda given the potential for the virus to be used as a biological weapon (71). For this reason and its ability to serve as a vector, vaccinia virus continues to be used in research. Murine models demonstrate that T_RM_ are generated in response to vaccinia and play a significant role in mediating protection against infection (72–76). Dermal-resident γδ T cells have also been implicated in the immune response against cutaneous vaccinia infection (77). Following skin infection CD8^+^ T cells are recruited independently of CD4 T^+^ cells and IFN-γ (72), many of which subsequently assume the T_RM_ phenotype (72, 73, 75, 78, 79) and are capable of initiating potent inflammatory responses upon re-stimulation (79). Of particular interest is the capacity of local vaccinia skin inoculation to globally seed skin tissue even at remote sites with long lasting T_RM_ (72) as well as generating T_RM_ responses in non-related non-lymphoid organs such as the lungs and liver (76). Multiple exposures to cognate viral antigens have also shown to selectively expand T_RM_ (72, 73, 78, 79). In a lung infection model of vaccinia, higher numbers of lung T_RM_ correlated with better protection against subsequent infection as indicated by a rapid reduction in viral loads. T_RM_ seem to expand more rapidly and localize to the infection site as indicated by a 5-ethynyl-2′-deoxyuridine proliferation assay when compared to their circulating counter parts. Depletion of lung CD8^+^ T cells by intranasal administration of αCD8 antibody, resulted in previously protected mice becoming susceptible to infection, indicating that CD8^+^ T_RM_ play a vital role in mediating immunity (74). In another study, parabiosis experiments demonstrated that T_RM_ were exceedingly better at clearing vaccinia virus skin infection than T_CM_ within a shorter timeframe. In fact, it appears that skin T_RM_ can clear vaccinia skin infection even in the absence of neutralizing antibodies and T_CM_ (72). However, vaccinia-specific CD8^+^ skin T_RM_ appear to have an impaired ability to recruit circulating effector cells during polymicrobial sepsis infection (80). Whether there are other physiologically challenging conditions that impair skin T_RM_ functionality remains largely unexplored. Surprisingly, vaccinia lung infection revealed that not all T_RM_ are equally capable of conferring protection. T_RM_ that resided in the lung interstitium were better positioned to rapidly kill infected lung cells in a contact-dependent manner when compared to T_RM_ situated in association with the tissue vasculature. Furthermore, T_RM_ found within the interstitium, unlike vascular-associated T_RM_, were able to up-regulate CD69 expression, potentially indicating an enhanced ability to respond during early infection (74). Investigations of vaccinia infection has also reinforced that epithelial immunization routes, such as skin scarification and intranasal exposure, demonstrate significant efficacy for generating protective T_RM_ responses (72–76, 78). In fact, vaccination *via* skin scarification is capable of protecting against clinical disease (pock lesions of the skin) whereas not all mice vaccinated *via* systemic routes such as intramuscular and intraperitoneal were protected from pock lesions. More astonishingly, mice immunized *via* skin scarification demonstrated greater resistance to disease when challenged *via* a heterologous route (intranasal), compared to mice immunized subcutaneously or intraperitoneally, in spite of generating reduced antibody titers (75). These observations may be attributed to T_RM_-mediated immunity given the evidence that T_RM_ can be generated in distant tissues after skin scarification (76). Overall, studies that use vaccinia infection models have shed light on the ability of skin scarification to elicit a robust and somewhat unique immune response.

## Respiratory Syncytial Virus (RSV)

Respiratory syncytial virus is a common cause of lower respiratory tract infections in children and the elderly. Common reinfection with RSV suggests absence of protective immunity (81). A number of studies have shown the importance of T_RM_ in providing protection against RSV (82–86). An experimental human infection study showed that adults with higher frequencies of RSV-specific CD8^+^ T cells, many of which displayed a T_RM_ phenotype, developed less severe lower respiratory tract symptoms and reduced viral loads. This increase in protection was not correlated with higher numbers of circulating CD8^+^ T cells, suggesting the localization of T_RM_ was vital for mediating immediate protection (82). T_RM_ induction in lung tissue and airway fluid was also demonstrated following intranasal RSV infection in mice. Adoptive transfer of airway lymphocytes from RSV-infected mice into naïve recipients reduced disease burden upon infection challenge, compared to adoptive transfer of airway lymphocytes from sham-infected mice. It was concluded that both airway CD8^+^ and CD4^+^ T cells play a role in protecting against RSV infection and reducing disease severity, respectively (83). However, given that only bulk CD4^+^ or CD8^+^ T cells were transferred, it remains to be investigated if the protective capacity is mediated by airway T_EM_ or T_RM_ cells. In support of the latter, an African green monkey model of RSV infection illustrated that antibody and CD4^+^ T cell responses are unlikely to protect against reinfection. On the contrary, it appears that lung CD8^+^ T cells, of which up to half displayed a T_RM_ phenotype, were more capable of protecting against secondary infection (84). From the available evidence (82–86), it appears that an ideal RSV vaccine should elicit a CD8^+^ T_RM_ response in the lung. Of note, some experimental RSV vaccines have already shown promising results with regards to T_RM_ generation: intranasal administration of an RSV antigen-expressing murine cytomegalovirus generated an IFNγ- and MIP-1β-secreting population of T_RM_ (85); co-administration of the TLR9 agonist CpG and an inhibitor of notch signaling (L-685,458) with formalin-inactivated RSV elicited a strong protective T_RM_ response (86); intranasal administration of virus-like particles containing RSV M and M2 proteins as antigen delivery systems has also shown propensity to induce the production of T_RM_ (87); and a dendritic cell-*Listeria monocytogenes* immunization strategy, when administered locally, was able to avoid circulating T cell-induced immunopathology and protect against RSV infection challenge through the generation of T_RM_ (88).

## Cytomegalovirus (CMV)

Cytomegalovirus establishes life-long latency in many organs including mucosal tissues. It has long been known that CMV infection induces a sustained clonal expansion of specific CD8^+^ T cells, a phenomenon referred to as memory inflation (89). However, only recently has it been explicated that CMV infection promotes the formation of T_RM_ in various mucosal tissues, especially the salivary glands (90–92). Although the CD8^+^ T cell response is vital for the control of CMV infection, the virus-induced downregulation of MHC I on acinar glandular cells of the salivary glands (long-term target tissue of CMV) resulting in the reliance on CD4^+^ T cells for control of lytic replication at this site (93). Surprisingly, salivary gland CD8^+^ T_RM_ were capable of controlling viral replication. It appears that murine CMV is unable to completely inhibit the expression of MHC I on CD8^+^ T_RM_ of the salivary glands, thereby providing an opportunity for these T cells to mediate localized immunity (90). Although it remains unclear whether these T_RM_ inhibit viral replication through effector cytokines or direct cytotoxicity, it certainly appears that salivary gland T_RM_ may inhibit the shedding of CMV, hence reducing the chances of transmission. These mucosal T_RM_ typically form early after infection. However, mucosal seeding continuously occurs through the recruitment and differentiation of circulating populations. As such, the immunodominance of mucosal T_RM_ against CMV changes with time, favoring the TCR repertoire that remains high in circulation (91). T_RM_ have also been found in brain tissue after murine CMV infection (94–96). In the brain, CMV-specific T_RM_ formation seems to be dependent on regulatory T cell (T_reg_) activity. Furthermore, T_reg_ cells seem to have a suppressive effect on brain T_RM_’s capacity to produce granzyme B, potentially a precautionary measure to prevent detrimental neuroinflammation (94). From the studies that have dissected the role of T_RM_ in protecting against CMV infection and inhibiting reactivation there seems to be a clear role for these tissue tropic T cells in limiting CMV replication. A number of studies also demonstrate the capacity of CMV to be used as a viral vector in novel vaccines that generate T_RM_-mediated immunity (85, 91, 97). Manipulating CMV’s capacity to induce a robust CD8^+^ T cell response within mucosal tissues may be a promising avenue for the generation of new vaccines.

## Lymphocytic Choriomeningitis Virus (LCMV)

Lymphocytic choriomeningitis virus, a rodent-borne disease can cause meningoencephalitis in humans (98). While LCMV infection models have been used to study T_RM_ in multiple tissues (99), the protective role of T_RM_ has only been clearly investigated in the brain, thymus, and female reproductive tract. Depletion of circulating T cells or NK cells demonstrated that T_RM_ have the capacity to protect against infection independently of NK cells, T_CM_, and T_EM_ populations (100, 101). Upon MHC-I-antigen stimulation, LCMV brain T_RM_ displayed effector functions and mediated virus control through IFN-γ release and perforin-mediated cytotoxicity (100). Thymic T_RM_, when stimulated with gp33, released both IFN-γ and TNF-α, suggesting that T_RM_ at this location may be polyfunctional. It also appears that T cells that took residence in the thymus were more likely to respond to antigen stimulation when compared to their splenic counterparts, further exemplifying the protective nature of these cells (101). Since infection of the thymus can significantly reduce T cell generation due to increased thymocyte deletion and reduced proliferation, it is vital to have protective mechanisms in place that act rapidly to minimize pathogen-induced damage in the thymus. From the available evidence, thymic T_RM_ seem to be capable of adequately fulfilling this task. LCMV infection also induces the production of T_RM_ in various peripheral tissues, such as the lungs, intestines, and female reproductive tract (102–104). While the role of T_RM_ in the lung and intestines following LMCV infection is not well established, it was found that re-activation of CD8^+^ T_RM_ in the female reproductive tract was able to produce a general anti-viral immune response that is almost able to confer sterilizing immunity when challenged with an non-cognate virus (105). This T_RM_ induced antiviral state may be of great interest in the aim to generate vaccines that create heterotypic protection.

## Varicella Zoster Virus

Varicella zoster virus, the cause of chicken pox, is an alpha-herpes virus that can establish latency within the dorsal root ganglia. Reactivation of the virus results in a painful disease called shingles. Although vaccines are available against both chicken pox and shingles (106), recent evidence suggests that T_RM_ may be key players in controlling latent infection, a phenomenon that could be exploited to improve current vaccines. One study analyzed skin samples from human donors of varying ages who were serologically confirmed VZV positive. 80–90% of T cells from the sampled tissue expressed CD69, suggesting that the majority of T cells in skin were T_RM_. IL-2 responses from stimulated VZV-specific T cells demonstrated that host age did not influence the numbers of responsive cells. However, it was found that skin from older donors demonstrated a lesser capacity to mount a clinical response and decreased CD4^+^ T cell infiltration when challenged with VZV antigen. This correlated with higher proportions of Foxp3^+^ cells. Furthermore, T_RM_ of older skin expressed PD-1 in higher amounts (107). Together, this data suggest that VZV-specific T_RM_ may be suppressed with age. This may be a reason for the high incidence of reactivation of VZV in older individuals. Results from a different study that utilized samples of human trigeminal ganglia suggests that T_RM_ do not seem to play a role in controlling latent infection in the trigeminal ganglia (10). Regardless, further investigation into the role of T_RM_ in controlling latent VZV infection may help to develop therapeutics or vaccines that prevent shingles.

## Human Papillomavirus

Human papillomavirus is a sexually transmitted pathogen that generally causes an asymptomatic, self-limiting infection. However, certain subtypes of HPV can cause cancer of the cervix, anus, and oropharynx (108). The routine administration of HPV preventative vaccines has led to a significant reduction in the incidence of infection in many parts of the world. However, immunization of individuals with an established HPV infection has not shown to protect against the progression of HPV-induced lesions into carcinoma. As such, a therapeutic vaccine that is administered by post infection may subvert this problem. Current HPV vaccines rely on the induction of antibodies to neutralize viral particles (109). The potential for generating anti-HPV T_RM_ as a strategy for eliminating previously established HPV infection is yet to be fully explored. One study evaluated the capacity of two adenoviruses (Ad26 and Ad35) that express a fusion of the HPV16 oncoproteins E6 and E7 to elicit a protective response in the cervicovaginal mucosa. Intra-vaginal administration of either vector was able to elicit the generation of CD8^+^ T_RM_ within the cervicovaginal mucosa. Furthermore, systemic priming with Ad35 followed by an intra-vaginal booster immunization of Ad26 induced polyfunctional, E6/E7-specific, cytokine-secreting CD8^+^ T cells within the cervicovaginal mucosa (110, 111). Although it remains to be resolved if protection against established HPV infection causally relies on T_RM_, this and other studies (111) provide impetus to further explore the intra-vaginal route of administration and the use of viral vectors as strategies for the induction of cervicovaginal T_RM_.

## Viral Hepatitis

Viral hepatitis is an inflammatory disease of the liver that is caused by a range of viruses (112). Two studies, both of which utilized human donor liver tissue and paired blood samples, analyzed the role of T_RM_ in the context of viral hepatitis. One study focused on patients with hepatitis B viral infections (HBV), while the other study included patients with HBV or hepatitis C viral infections. A higher proportion of liver T cells from patients who demonstrated partial control of HBV infection had a T_RM_ phenotype, when compared to healthy controls. Given that the overall numbers of T cells in the liver of healthy and HBV-infected individuals were similar, this threefold increase in T_RM_ numbers appear to be due to an increased predisposition of T cells to adopt the T_RM_ phenotype in virally infected liver tissue, rather than expansion of pre-existing T_RM_ (113). The numbers of T cells co-expressing CD69 and CD103 increased by fourfold in chronic hepatitis C patients (114). Furthermore, the reciprocal relationship between viral loads and liver T_RM_ numbers indicates that T_RM_ play a vital role in infection control (113). *Ex vivo* stimulation of T_RM_ showed heterogeneous antigen specificity, with a number of HBV antigens being able to initiate effector responses. However, viral envelope peptides seemed to generate the greatest capacity to induce production of IFNγ, TNFα, and IL-2. Analysis of T_RM_ from healthy liver tissue revealed a noticeably reduced expression of granzyme B, when compared to non-resident counter parts. This suggests that hepatic T_RM_ have less cytolytic capacity than circulating T cells (113, 114). However, liver T_RM_ of patients with chronic hepatitis B expressed markedly higher amounts of granzyme B when compared to healthy controls (114). Liver T_RM_ also showed increased expression of the inhibitory molecule PD-1 compared to non-resident T memory cells (113, 114). The downregulation of granzyme B and upregulation of PD-1 in healthy liver tissue may be a precautionary measure intended to prevent immunopathology, given the liver’s role in filtering high amounts of antigen draining from the mesenteric circulation. This is of great importance in viral hepatitis infections as immunopathology is largely involved in the progression of viral hepatitis that leads to cirrhosis and hepatocellular cancer. The increased production of granzyme by T_RM_ in CHB patients may be part of the pathogenesis of fulminant hepatitis. Further exploring the role of T_RM_ in protection against viral hepatitis (including hepatitis A, D, and E) and the immunopathology implicated in the progression of the disease may aid in the development of immunomodulatory therapeutics to prevent viral cirrhosis and hepatocellular cancer.

## Epstein–Barr Virus (EBV)

Epstein–Barr virus is one of the most prominent causes of infectious mononucleosis. After exposure to infected saliva, the virus infects and replicates in B cells and epithelial cells of the new host. Although the clinical disease of glandular fever is usually self-limiting, EBV remains latent in circulating B cells and episodes of reactivation are known to occur. It appears that reactivation of EBV occurs in the lymphoid tissue of the oropharynx, where the virus switches from a latent form into a lytic cycle. Control of infection is mediated by a T cell response against infected B cells (115). EBV-specific CD8^+^ memory T cells localize to the epithelium of the oropharynx (116), where they up-regulate CD69 and CD103 in an IL-15- and TGF-β-dependent fashion (117). CD103^+^ EBV-specific T memory cells found in tonsillar tissue are more sensitive to antigen stimulation and produce a greater effector response when compared to circulating EBV-specific T cells (116). Furthermore, a substantial CD103^+^ T cell population only seems to appear as viral replication and disease tapers (118). Taken together, it appears as though T_RM_ play a crucial role in rapidly controlling viral replication of EBV within the oropharyngeal tissue upon reactivation to prevent full clinical relapse.

## Vesicular Stomatitis Infection (VSV)

Vesicular stomatitis infection is a zoonotic disease that can cause a mild febrile illness in humans (119). Intranasal infection of mice with VSV has shown to produce CD103^+^ CD8 T_RM_ population in the brain (120, 121), as the virus travels along the olfactory bulb to the brain where it causes infection. These brain T_RM_ were found to be functional *in situ*, responding to cognate antigen (120, 121). Staining for effector molecules revealed that many of these T_RM_ cells were positive for granzyme B, suggesting cytolytic abilities. Once removed from the brain parenchyma, these cells appear dysfunctional, suggesting they are highly adapted to the brain microenvironment. Maintenance of this population of T_RM_ appears to be independent of circulating T cells, and BrdU incorporation indicates a slow homeostatic rate of proliferation to sustain the population (120). Interestingly, brain T_RM_ appear to form clusters within specific sites of the brain parenchyma that contain CD4^+^ T cells, perhaps indicating a role for CD4^+^ T cells in the generation and/or maintenance of brain CD8^+^ T_RM_. These clusters may have formed around sites of previous VSV replication sites, where persisting antigen may be drawing the T_RM_ to these locations. Although, viral RNA could not be detected at these sites (120), this does not exclude the possibility that undetectable levels of antigen may be present at these sites. T_RM_ may also form clusters around local dendritic cells that are still presenting antigen from a previous infection. This hypothesis is supported by the observation that antigen presentation by bone marrow-derived-APCs was able to support CD103 expression by T_RM_ (120).

## Other Viruses

Polyomaviruses are opportunistic pathogens that usually remain latent following infection. However, in immunocompromised individuals, infection can cause multifocal leukoencephalopathy (122). T_RM_ are generated in the context of polyomarvirus infection (123–126), and polyomavirus-specific brain CD8^+^ T_RM_ in mice maintain a high TCR affinity for pathogen epitopes. In fact, T_RM_ TCR affinity appears to be higher than the TCR affinity of T cells from the spleen. This observation supports a role of T_RM_ in mediating rapid control of viral replication during reactivation, as high TCR affinity allows for the early detection of low amounts of virus (123). In contradiction to this finding, evidence from another study suggests that lower TCR stimulation increases the generation of brain T_RM_ (125). One way of interpreting these seemingly contradicting observations is that brain T_RM_ initially differentiate from circulating effector T cells with low TCR stimulation capacity, but after taking residence in the brain, undergo functional avidity maturation (127) increasing their ability to respond to antigen. A renal transplant clinical study suggests that renal BK Polyomavirus-specific T_RM_ were rendered incapable of protecting against infection leading to interstitial nephritis, likening these T_RM_ to dysfunctional tumor-infiltrating lymphocytes (126).

Ebola virus causes a form of hemorrhagic fever characterized by intravascular coagulation and maculopapular rash. Although the natural reservoirs for the virus are thought to be fruit bats, human-to-human transmission can occur when contaminated body fluids breach mucosal barriers or skin. Absence of specific treatment and epidemic potential of the virus highlights the need for a vaccine (128). Aerosol administration of a human parainfluenza virus type 3-vectored vaccine expressing an Ebola envelope glycoprotein was capable of not only eliciting neutralizing antibodies but also a CD103^+^ T cell response in the lungs of macaques. A large proportion of these T_RM_ were polyfunctional, demonstrating positivity for two or more activation markers. Furthermore, a single dose of this vaccine conferred 100% protection against infection challenge (129). Since a large proportion of transmission in the recent Ebola epidemic was through skin contact, vaccination *via* scarification is worth exploring.

Norovirus is a highly infectious virus, and is a common cause of gastroenteritis. Although infection is generally self-limiting, chronic forms have been reported in immunocompromised patients. A clinical study has implicated CD8^+^ T cells resembling T_RM_ in the immune response against norovirus (130). However, a genetically manipulated strain of murine norovirus causing chronic infection revealed that despite a robust and functional T_RM_ response being generated, clearance of the virus was not achieved, likely due to inadequate antigen sensing (131).

## The Role of T_RM_ in Bacterial Infections

Although there is significantly less literature about T_RM_ in the context of bacterial infections, the evidence largely implies that T_RM_ have a noteworthy role in protecting against pathogenic bacteria. The following section groups bacterial pathogens together depending on their location of primary infection.

## Bacterial Infections of the Lungs and Airways

Pertussis, also known as whooping cough, is caused by *Bordetella pertussis*, a Gram-negative coccobacillus. Despite high vaccination coverage, whooping cough remains a serious public health concern. T cell responses are critical for immunity against *B. pertussis* (132). While the existing whole-cell pertussis (wP) vaccine is generally associated with a strong Th1 response, immunization with the widely used acellular pertussis (aP) vaccine induces a Th2-dominated humoral response (133). Immunity to the aP vaccine wanes over time compared to wP vaccines (134). This diminished immunity allows for the transmission of *B. pertussis* to susceptible individuals. A recent study reported that following *B. pertussis* infection, IL-17- and IFN-γ-secreting CD4^+^ T_RM_ congregate in the lungs of infected mice where they persisted for 120 days, and expanded up to sixfold upon reinfection. Egress inhibitor FTY720 did not affect the control of bacterial burden during secondary infection, suggesting that T_RM_ were capable of providing immunity irrespective of peripheral T cell recruitment. Bacterial clearance in reinfected mice also correlated with CD4^+^ T_RM_ expansion, with a large portion of cells displaying a Th17 phenotype (135). Adoptive transfer of lung CD4^+^ T_RM_ from infected mice into naïve hosts conferred protection against *B. pertussis* challenge (135), suggesting that Th17-like CD4^+^ T_RM_ seemed to play a crucial role in long-term immunity. Interestingly, γδ T cells that express CD69 and CD103, classically known to provide innate-like protection during primary infection, also provided a significant early-release IL-17 response during secondary infection in convalescent mice. However, γδ T_RM_, especially Vγ4^+^ γδ T cells persisted in the lungs of convalescent mice and produced a greater IL-17 response on re-exposure to *B. pertussis* in an antigen-specific manner (136). Therefore, a long-lasting *B. pertussis* vaccine should not only promote the generation of *B. pertussis*-specific CD4^+^ T_RM_ but also γδ T_RM_.

Pneumonia is one of the largest infectious causes of mortality in children worldwide (137). The most common cause of community-acquired pneumonia is *Streptococcus pneumoniae*, a Gram-positive polysaccharide-encapsulated bacterium (138). Modern pneumococcal vaccines are polysaccharide based and are thus poorly immunogenic, providing serotype-specific immunity that wanes over time. Although CD4^+^ Th17 responses are considered vital in providing protection against pneumococcal infections, the role of T_RM_ is yet to be fully characterized. Experimental *S. pneumoniae* infection was found to promote the production of heterotypic CD4^+^ T_RM_ of both Th17 and Th1 phenotypes in niches located within pneumonia-affected lobes of the lung. It was also observed that immunity was restricted to pathogen-experienced tissue, suggesting that T_RM_ reside in primary infection sites, rather than providing immunosurveillance throughout the entire respiratory mucosa. Despite spatial restriction, T_RM_ provided superior protection to the local tissue when compared to systemic immune responses elicited by antigen-specific CD4^+^ T_CM_. Neither adoptive transfer of splenic CD4^+^ T cells from infected mice into naïve recipients, nor inhibiting lung translocation of circulating CD4^+^ T cells with FTY720 in pathogen-experienced mice, had a significant effect on protection against pneumococcal infection challenge. Therefore, protective immunity against bacterial pneumonia is likely due to the aggregation of CD4^+^ T_RM_ in susceptible tissues (139). Interestingly, combining whole virion influenza and whole cell pneumococcal vaccine also promoted the generation of lung CD4^+^ T_RM_. It is likely that these T_RM_, in combination with the accompanying high antibody titers elicited by the combined vaccine, played a role in providing protection against pneumococcal-influenza co-infection (140). Overall CD4^+^ T_RM_ may play a role in the generation of naturally acquired immunity against pneumococcal infections, and should be considered in the development of heterotypic pneumococcal vaccines.

*Mycobacterium tuberculosis* (*Mtb*), an acid-fast staining intracellular bacterium, is the causative agent of tuberculosis (TB). The deadly infection can present as pulmonary, as well as extra pulmonary disease (141). Currently, *Bacillus Calmette–Guérin* (BCG) is the only licensed vaccine against TB, and prevents dissemination in children. However, BCG does not provide strong enough immunity against pulmonary TB in adults, therefore, allowing transmission (142). Immune control of *Mtb* infection largely relies on the production of IFNγ by CD4^+^ T cells, which enhances macrophage killing of persisting intracellular *Mtb* and leads to the formation of granulomas around sites of bacterial replication (141). A clinical study revealed that individuals previously exposed to tuberculosis were likely to have a population of lung-resident Th1 effector memory cells that released IFN-γ in response to *Mtb* antigen re-exposure (143). However, the delay in activation and recruitment of TB-specific T cells to the lungs during primary infection allowed for *Mtb* to proliferate, resulting in a high bacterial burden. The importance of airway-residing memory T cells (then called airway luminal cells) in mediating protection against TB has been described well before the dawn of T_RM_ (144–146). However, these cells most likely represent the same cell type. Lung T_RM_ induced by mucosal vaccination have shown to be effective in limiting the early control of bacterial replication (147). Despite the defined role of CD4^+^ T cells in controlling TB, recent evidence from vaccine studies suggest that CD8^+^ lung T_RM_ also play an important role in protection against *Mtb* (148, 149). Only mucosal administration of BCG led to the generation of airway T_RM_ that produce higher levels of pro-inflammatory cytokines, including IFN-γ than CD8^+^ T_EM_. Furthermore, adoptive transfer of sorted airway CD8^+^ T_RM_ from BCG-vaccinated mice demonstrated enhanced protection against *Mtb* challenge in recipient mice. Transfer of CD8^+^ T_RM_ decreased the numbers of alveolar macrophages, while increasing the number of CD4^+^ T cells and B cells in the infected lung tissues (148). It was hypothesized that CD8^+^ T_RM_ kill *Mtb*-infected alveolar macrophages, thereby depleting intracellular reservoirs of the bacteria and limiting the entry into the lung parenchyma (148). Likewise, the viral-vectored vaccines SeV85AB and AdAg85A, administered *via* the intranasal route have also shown to elicit an immune response that favors the production of CD8^+^ rather than CD4^+^ T_RM_ (149, 150). In a rhesus monkey model, a cytomegalovirus vector delivering a range of *Mtb* antigens (RhCMV/TB) provided significant protection against tuberculosis, presumably through it its ability to generate and maintain pathogen*-*specific CD4^+^ and CD8^+^ circulating and more importantly resident memory T cells that selectively express VLA-1 (151). Finally, aerosol vaccination with an attenuated *Mtb* strain lacking *sigH* not only led to an enormous influx of T cells expressing CD69 into the lung airways (likely to include T_RM_), but also to a significant long-term protection against virulent *Mtb* challenge (152). Collectively, these studies indicate that rationally designed TB vaccines should generate immune responses that prevent the establishment of infection and/or provide sterilizing immunity by inducing both lung CD4^+^ T_RM_ and CD8^+^ T_RM_ in the lungs.

## Bacterial Infections of the Urogenital Tract

A number of bacteria cause disease of the reproductive tract and urinary system. One such example is *Ct*, an obligate intracellular bacterium that causes infections of the genitals and eyes. It is the leading cause of infectious blindness worldwide, and can cause infertility when sexually transmitted (153). According to clinical evidence, it appears that spontaneous clearance of clinical infection correlates with at least partial protection against *C. trachomatis* through the production of INF-γ-secreting cells such as CD4^+^ Th1 cells. However, IFN-γ responses alone do not seem to provide complete protection. It has also been documented that B cell-antibody responses are involved in immunity, especially against secondary infection (154–156). Importantly, intraepithelial CD8^+^ lymphocytes and MLCs composed of B cells and CD4^+^ T cells border the vaginal and uterine tract, respectively in pathogen-experienced tissue. These immunocyte structures hinder *C. trachomatis* from replicating and establishing a clinical infection (157). Optimal protection against *Chlamydia* requires both the recruitment of T_CM_ and the presence of T_RM_ within the urogenital tract (158, 159). It is likely that protective immunity occurs in response to chronic or repeated infection, which leads to the seeding of T_RM_ throughout the epithelial surface (160). A vaccine composed of *Chlamydia* major outer membrane protein and ISOCMATRIX adjuvant was able to provide enough protection to prevent the sexual transmission of *C. trachomatis*, however, was not capable of providing complete immunity. This may be attributed to the inability of the vaccine to generate a large enough T_RM_ population, underscoring the essential role of T_RM_ in *Chlamydia* infection (159). In a separate study, mice were either inoculated with infectious *C. trachomatis* or UV-inactivated *C. trachomatis* (UV-*Ct*). Mice infected with the infectious form demonstrated capacity to control future infections better than naïve controls, which may be attributable to the production of both *Chlamydia*-specific T_CM_ and T_RM_ populations. However, the group of mice inoculated with UV-*Ct* suffered higher bacterial burdens when compared to naïve controls. This data in conjunction with the generation of *de novo Ct*-specific T_reg_ suggest that a tolerogenic immune response occurred in these mice. On the contrary, intra-uterine administration of UV-*Ct* conjugated with charge-switching synthetic adjuvant peptides (UV-*Ct-*cSAP) conferred a superior protection to *Ct* in both conventional and humanized mice. The rapid clearance of *Ct* in UV-*Ct-*cSAP-vaccinated mice has been attributed to the immediate release of IFN-γ by mucosal T_RM_ (158). Taken together, the ideal vaccine against *Chlamydia* should promote the generation of local MLC, T_CM_, and T_RM_ in the epithelium, even though partial protection appears to be sufficient to prevent disease transmission.

## Bacterial Infections of the Gastrointestinal Tract

Gastrointestinal infections are generally acquired through ingestion of contaminated food or water. These bacteria may remain in the gut, or may disseminate to other parts of the body causing systemic disease (161). Targeted induction of T_RM_ along the gastrointestinal epithelium could enhance protection against these pathogens. *Listeria monocytogenes*, a food-borne Gram-positive coccobacillus, is of particular concern in immunocompromised and pregnant individuals, and can cause meningitis and stillbirth (162). Its capacity to replicate within host cells facilitates immune evasion. Thus, protection against *L. monocytogenes* is largely dependent on cell-mediated immunity (163). First observed in 1981 as a distinct population of long-lived T memory cells “positioned” in tissue following listeriosis (164), a more recent study highlighted the role of intestinal CD8^+^ T_RM_ in mediating immunity against *L. monocytogenes* following oral infection. In fact, blockage of integrin α_4_β_7_ prevented the formation of these intestinal TGF-β-dependent T_RM_ resulting in diminished protection upon re-challenge (165). Revealed by multi-photon dynamic microscopy, a population of Vγ4^+^ γδ T_RM_ was found within the mesenteric lymph nodes in response to *L. monocytogenes* infection, which remained largely stationary under homeostatic conditions. However, upon re-challenge, activation of these cells resulted in organized clusters around bacterial replication foci where they released IL-17 and subsequently, the recruitment of neutrophils to facilitate bacterial elimination. Similarly to γδ T_RM_ function seen in *B. pertussis* infection (136), neutralization of IL-17 hindered bacterial clearance, highlighting the importance of early IL-17 release by γδ T_RM_ (166).

*Yersinia pseudotuberculosis* (*Yptb*), a food-borne pathogen causing of Far East scarlet-like fever, is a Gram-negative bacterium responsible for gastroenteritis, mesenteric lymphadenitis, and can clinically mimic acute appendicitis (167). A *Yptb* oral infection mouse model showed a robust CD8^+^ T cell response in the intestines including a population of *Yptb*-specific CD103^+^ CD8^+^ T_RM_ uniformly distributed throughout the intestine, while CD103^−^ T_RM_ formed around sites of primary infection where they carried out effector functions (168). Although found in antigen-rich areas, their development is independent of local antigen stimulation (168). The development of T_RM_ populations in the intestine seems to rely on inflammatory signals from the site of infection rather than antigens (169). Production of IFN-β and IL-12 from intestinal macrophages effectively suppresses TGF-β-mediated CD103 expression thereby leading to the development of CD69^+^ CD103^-^ T_RM_ population in mice during *Yptb* infection. This data suggest a central role for inflammatory monocytes in the differentiation and maintenance of different CD8^+^ T_RM_ populations to achieve optimal protection against intestinal infections. Additionally, this study raises the question as to whether CD103 is necessary for residence within the intestinal tissue, or whether it negatively regulates T_RM_ capacity to migrate within their residential tissues.

*Salmonella* spp. is a group of Gram-negative bacilli that is a common cause of gastrointestinal infections responsible for “food poisoning.” Transmitted orally, this heterogeneous group of bacteria contains typhoid or enteric-fever causing serovars that can be potentially fatal to humans (170). Current vaccines against *Salmonella* are poorly immunogenic and risk disease in immunocompromised individuals (171). An effective vaccine that prevents gastrointestinal infection is much needed to prevent outbreaks of salmonellosis. Subcutaneous co-administration of *Salmonella* SseB and flagellin has shown to provide protection against systemic disease in mice. However, parabiosis studies suggest that this protection can be transferred *via* the circulation, diminishing the role of T_RM_ in the observed immunity (172). Nevertheless, it may be beneficial to assess the capacity of this vaccine and others to protect against gastrointestinal infection by oral administration. However, the barriers of oral tolerance and destruction of vaccine components by digestive enzymes and chemicals must be overcome in order to develop oral vaccines.

In summary, T_RM_ responses in bacterial infections appear to be more diverse compared to viral infections, an observation that may be attributed to the varying locations of bacterial replication (intracellular versus extracellular), the more complex lifestyles and the presence or more sophisticated immune evasion mechanisms. Future studies should assess the role of T_RM_ in the natural immune response to other bacterial infections.

## The Role of T_RM_ in Parasitic (Protozoa and Helminths) Infections

Protozoa are unicellular organisms that are of great importance to human health. Most prevalent in tropical regions of the world, protozoan infections are difficult to treat due to their complex life cycles and their ability to evade host immune responses through antigenic variation, residence within various intracellular compartments, and their capacity to assume protective forms such as cysts (173, 174). At this juncture, our collective understanding of T_RM_ responses to protozoan infections remains relatively deficient. However, studies have shown that T_RM_ play a significant role in protecting against a few protozoan species.

Malaria, the most prevalent protozoan infection of humans, is caused by five species of *Plasmodium*. Transmitted by the bite of an infected female *Anopheles* mosquito, *Plasmodium* parasites enter the circulation and take residence inside erythrocytes during part of their complex life cycle. Natural immunity to *Plasmodium* infection involves a mixture of humoral, CD4^+^, and CD8^+^ T cell responses (175). However, liver T_RM_ have emerged as a promising target for protecting against malaria (176). Unlike T_RM_ of the epithelium, such as the lungs, intestines, and skin, liver-T_RM_ appear to reside in sinusoids (the blood vessels of the liver), rather than the parenchymal tissue (177–179). The heavily fenestrated architecture of these blood vessels and the distinct slow flow rate of blood allows for T_RM_ to traverse through the organ without being dispatched into circulation. Furthermore, liver sinusoids provide a prime niche for close interaction of T_RM_ and antigen presenting cells, such as Kupffer cells and dendritic cells. This allows for the rapid detection of antigen. Each hepatocyte is also in close association with a sinusoid, thereby providing easy access to liver T_RM_ for assessment of surface antigen presentation (177). Intravital imaging revealed that these T_RM_ traversed around 10 µm per minute and, as reported in HSV-1 infection of skin, T_RM_ assume an amoeboid form, extending dendrites to survey the liver for antigens (2). Rather than relying on CD103-αE integrin interactions for maintaining tissue residence, liver T_RM_ appear to utilize the adhesion molecule LFA-1 (3). A rhesus monkey *P. knowlesi* infection model that assessed sporozoite immunization demonstrated capacity for generating liver T_RM_. These T_RM_ appear to be protective as their depletion resulted in the loss of immunity (1). Experiments with radiation-attenuated sporozoites also support the notion that inducing high numbers of liver T_RM_ can afford protection against malaria. In this study, a “prime-and-trap” strategy was used in which primed T cells from the spleen were drawn to the liver using a recombinant adeno-associated virus that infected hepatocytes and subsequently caused them to express *Plasmodium* antigen. The immunity generated by this strategy was attributed to the increased numbers of CD8^+^ liver T_RM_ (2). The emergence of liver T_RM_ as a potential target for pre-erythrocytic malaria vaccines warrants further research.

Leishmaniasis is a heterogeneous vector-borne disease that is caused by an intracellular protozoa parasite. There are over 20 known *leishmania* species, all of which are transmitted by bites from infected female *Phlebotomine* sandflies. Clinical disease presents in three main forms: cutaneous, mucocutanous, and visceral. Subjugation of *Leishmania* parasites relies on the establishment of IFN-γ-producing CD4^+^ Th1 cells (180). While it is widely known that clearance of primary infection can lead to protective immunity, the effector response that leads to protection remains unclear. It has, however, become apparent that T_RM_ play a crucial role in providing such protection (181–183). *L. major* infection models illustrated that following infection, long-lived T_RM_ are rapidly fabricated and seeded universally throughout the skin, as they can be detected in tissue far away from the primary site of infection (181). It was previously believed that these T_RM_ provided immunity by rapidly recruiting circulating effector T cells. However, more recent studies suggest that the recruitment of circulating T cells may not be as important as previously thought, as FTY720 and αCXCR3-treated mice re-challenged with *L. major* showed minimal difference in early parasite control (182, 183). Furthermore, parabiosis studies demonstrated that *Leishmania*-specific circulating T cells alone provide little or no protection during early infection. Data exemplifies that CD4^+^ T_RM_ are rather likely to provide immunity by eliciting a delayed-type hypersensitivity response. Early immunity is attributable to the capacity of T_RM_ to rapidly recruit reactive oxygen species/nitric oxide producing inflammatory monocytes to control parasite burden (182). Liver T_RM_ have also been implicated in the immune response against *Leishmania*. The recombinant proteins LirCyp1 and LirSOD of *L. infantum* appear to be good candidates for promoting the expansion of liver memory T cells (184). No current vaccine exists for this potentially fatal disease. Together, these data suggest that Leishmaniasis vaccines should be tailored to generate T_RM_ to provide heterotypic protection against the many species that cause disease.

*Toxoplasma gondii*, the causative agent of toxoplasmosis, is an intracellular protozoan that is generally acquired through contaminated food. In humans, *T. gondii* can form persistent cysts in multiple tissues (185). In both acute and chronic infection, cell-mediated immunity and effector cytokines play vital roles in limiting the progression of disease (186). Mice deficient in TNF-α suffer from increased pathology, and IFN-γ production in the brain stimulates microglia and astrocytes to inhibit protozoan proliferation. In a chronic infection model of *T. gondii*, CD103^+^ CD8^+^ T_RM_ established in the brain produced a more robust IFN-γ and TNF-α response when compared to CD103^−^ T cell subsets (187). It appears that brain T_RM_ provide superior protection against *T. gondii* infection of the central nervous system when compared to CD8^+^ T_EM_ and T_CM_. During *T. gondii* and *Y. pseudotuberculosis* infection T_RM_ also seem to accumulate in white adipose tissue in what appears to be a depot of protective memory cells (188).

Helminthic infections are highly prevalent around the world. Although most infections are not fatal, they account for a large proportion of disease burden, causing secondary conditions, such as anemia and malnutrition. Immunity against helminthic infection is largely mediated by the Th2 effector arm of the adaptive immune system (189). The role of T_RM_ in protecting against helminthic infections, however, has only been explored recently in two species: *Heligmosomoides polygyrus* and *Nippostrongylus brasiliensis* (190, 191). While neither of these species are human pathogens, they provide analogous models to gastrointestinal helminthic infections and *Necator americanus* infection in humans, respectively (192, 193). Adoptive transfer of peritoneal-cavity CD4^+^ T_RM_ from convalescent mice into naïve mice prior to *H. polygrus* infection challenge, has demonstrated that peritoneal-cavity derived CD4^+^ T_RM_ are capable of hindering the reproductive capacity of female worms without reducing worm burden (190). This phenomenon provides new insight into what appears to be a unique interaction between T_RM_ and pathogen. A different study that used a *N. brasiliensis* model demonstrated that even a small number of lung-interstitial T_RM_ were capable of providing protective immunity. This was confirmed as cognate mice treated with FTY720 and lymphotoxin beta-receptor fusion protein (which causes lymphopenia) were able to clear secondary infection, suggesting that circulating T cells are not necessary to mount a protective secondary response (191). In spite of the lack of knowledge surrounding the interaction between T_RM_ and helminths, there is a clear role for this subset of T cells in worm infections that needs to be explored further.

## The Role of T_RM_ in Fungal Infections

Typically, fungal infections are less frequent compared to viral and bacterial diseases. However, due to the increasing use of immunomodulatory drugs for cancer and organ transplant patients, the increasing incidence of mycosis is of clinical importance (194). T_RM_ responses are least studied in the context of fungal infections. In fact, only one fungus appears to have been used in T_RM_ studies.

*Candida albicans* is a dimorphic yeast and opportunistic pathogen. Although it forms part of the normal commensal biome of humans, it can cause infections known as candidiasis especially in immunocompromised individuals (195). Skin and tongue-resident CD4^+^ IL-17-producing T_RM_ can provide effective protection against *C. albicans* (196, 197). Murine skin and oral infection models demonstrated that during early infection, γδ T cells release IL-17 in response to *C. albicans* invasion (196, 197). In skin, by day 7 post-infection, the vast majority of IL-17-producing cells are of the Th17 phenotype. Eventually, the T cells at the site of initial infection upregulate CD103 and CD69 suggesting they assume the CD4^+^ T_RM_ phenotype between 30 and 90 days post infection. During this time, the cells first become less motile, eventually “sessile” and localize to the papillary dermis. However, upon reinfection, these T_RM_ were capable of rapidly clearing infection and appear to be superior at doing so than circulating T_EM_. It was also reported that *C. albicans*-specific Th17 cells were found in high numbers in normal human skin (196), and that low doses of *C. albicans* antigen exposure stimulates the production of regulatory skin T_RM_ that substantially suppress the activity of skin T_EM_ (198). This may be attributed to the widespread presence of *C. albicans* in human tissue. Resident memory Treg cells may play a protective role in preventing a hyper-inflammatory response to benign *C. albicans* antigen exposure. Although the development of a vaccine against *C. albicans* is not of vital importance, these studies provide an initial insight into understanding T_RM_ responses to fungal infections. Other fungal infections that would be of interest include tinea, cryptococcosis, and aspergillosis.

## Discussion

**Figure 3:**
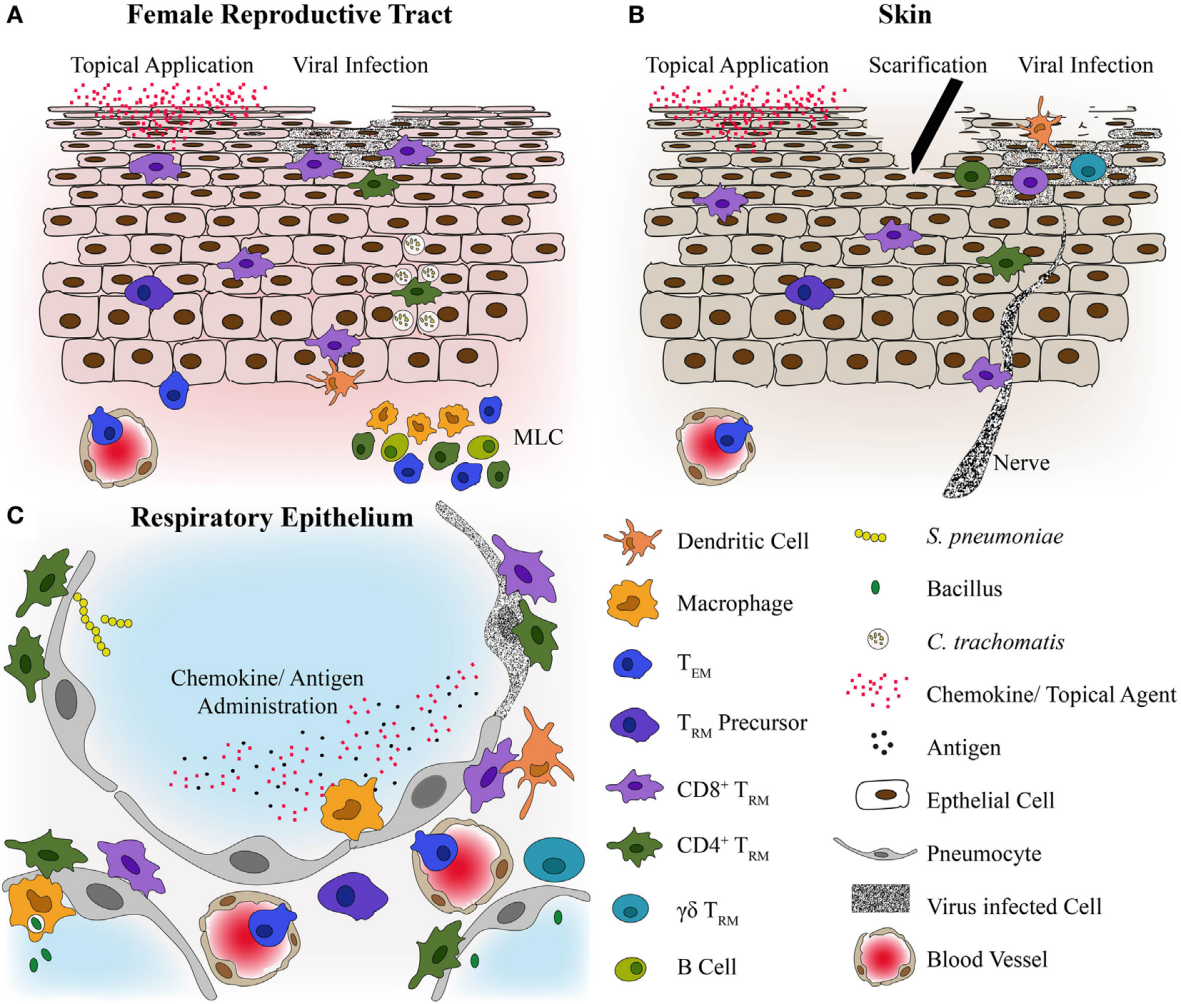
Visual summary of key T_RM_ effector responses and vaccine strategies at epithelial surfaces. **(A)** Represents the female reproductive tract. Topical application of both specific chemokines and general inflammatory agents such as nonoxynol-9 can be used to “pull” systemically primed T_EM_ into the mucosal tissue. CD103^+^ T_RM_ reside closer toward the apical surface of the mucosa. Both CD4^+^ and CD8^+^ T_RM_ play a role in controlling viral infections. Memory lymphocyte clusters have been shown to be important in controlling infections at this site. CD8^+^ T_RM_ re-stimulation appears to be dependent on CD301b^+^ dendritic cells that reside in the lamina propria. **(B)** Represent the integumentary epithelium. Topical application of both specific chemokines and general inflammatory agents such as 2,4-dinitroflourobenzene can be used to “pull” systemically primed T_EM_ into the epidermal tissue. Skin scarification as a route of vaccination encourages the development of skin T_RM_. Upon antigen recognition, skin T_RM_ lose their dendricity and become less motile. γδ T_RM_ can mediate early immune responses. CD8^+^ αα^+^ T_RM_ have been found in the dermal–epidermal junction where they may be able to survey local neural tissue for reactivation of latent viral infections. **(C)** Represents the respiratory epithelium. While different chemokines have shown the ability to “pull” T_RM_ into the respiratory epithelium and airways, the presence of antigen appears to be important at this site. T_RM_-mediated control of *Streptococcus pneumoniae* is largely dependent on the CD4^+^ subset. Control of viral and *Mycobacterium tuberculosis* infection requires both CD4^+^ and CD8^+^ T_RM_. γδ T_RM_, in conjunction with CD4^+^ T_RM_ have been shown to mediate immunity against *Bordetella pertussis*.

The discovery of T_RM_ has enhanced the possibility to develop new and improved vaccines. From the available literature, it appears that the most important factor for generating T_RM_ is to match the route of vaccination to the route of pathogen entry. In general, these are the mucosal and epithelial barriers that provide the first line of defense against pathogens: the respiratory, gastrointestinal, urogenital mucosa, and integumentary epithelium (Figure 3). Thus, the long-standing method of administering vaccines parenterally may be less effective at conferring optimal protection when compared to the novel mucosal and epithelial vaccine strategies highlighted throughout this review. The “prime and pull” method in which a parenteral vaccine is administered (prime) and an inflammatory agent is applied at a later time point to the desired peripheral tissue (pull) has also proven itself to be an effective vaccine strategy for generating T_RM_. The combination of mucosal vaccination and “pull” strategies may be an avenue worth exploring in future experiments. Another promising vaccine strategy is the use of viral-vectored vaccines. Regardless, it is also imperative to keep in mind the balance that these tissues must constantly strike in inducing a tolerogenic versus effector immune response to antigens, given their dual function in both their physiological roles (respiration, digestion, reproduction, etc.) and in serving as barriers against infection. Additionally, it will be important to consider the local cytokine milieu that influences T_RM_ generation (199). Generating memory T cells in peripheral tissues with cytolytic effector functions and the capacity to recruit inflammatory cells may pose the risk of inducing a hyper-inflammatory state leading to immunopathology. However, the natural persistence of T_RM_ in most peripheral tissues for extended periods of time under homeostatic conditions implies that mechanisms are in place to regulate T_RM_ responses. Developing a greater understanding of these mechanisms is vital in creating safe T_RM_-based vaccines.

A very appealing aspect of T_RM_-based vaccines is that it may be possible to generate heterotypic protection, as shown by studies using influenza infection models. This is vital for protecting against a range of rapidly mutating pathogens, such as HIV, as well as infectious diseases such as leishmaniasis that are caused by heterogeneous pathogens. Furthermore, the promptitude with which T_RM_ mediate immune responses is also of great interest when generating protection against infections such as tuberculosis that are capable of establishing latent infections. In order to achieve immediate T_RM_ protection, it appears that there must be a minimal density of T_RM_ within peripheral tissues to ensure pathogens are identified and eliminated in a timely manner. This may pose a challenge in developing T_RM_-based vaccines as sufficient numbers of T_RM_ need to be generated and maintained evenly throughout infection-susceptible tissues. The spatial capacity in non-lymphoid tissue to accommodate for T_RM_ may be limited, and thus establishing the capacity of different tissues has as well as determining the minimum threshold of T_RM_ needed to provide protection is much needed.

## Conclusion

In summary, the findings of this review largely accentuate the importance of T_RM_ in protecting against a range of pathogens. Their localization to sites prone to infection appears to give T_RM_ an enhanced capacity to mount swifter immune responses when compared to circulating memory T cells. Previous vaccine development has been largely centered on the generation of systemic memory response, which at times has shown to be ineffective. The capacity to form tissue-specific immunity through T_RM_ may shape vaccines of the future. Continuing to foster the growing pool of knowledge about T_RM_ will help to guide the field of T_RM_ vaccinology and may lead to the generation of new and more effective vaccines which may help to reduce the incidence of many infectious diseases.

## Author Contributions

VM performed the literature search and conducted extraction of data from relevant studies. AK, HS, and SN critically reviewed the literature search. VM and AK wrote the manuscript. All coauthors read and approved the final version of the manuscript.

## Conflict of Interest Statement

The authors declare that the research was conducted in the absence of any commercial or financial relationships that could be construed as a potential conflict of interest. The handling Editor is currently co-organizing a Research Topic with one of the reviewers ET, and confirms the absence of any other collaboration.
